# Expansion of the genetic toolkit for metabolic engineering of *Clostridium pasteurianum*: chromosomal gene disruption of the endogenous CpaAI restriction enzyme

**DOI:** 10.1186/s13068-014-0163-1

**Published:** 2014-11-19

**Authors:** Michael E Pyne, Murray Moo-Young, Duane A Chung, C Perry Chou

**Affiliations:** Department of Chemical Engineering, University of Waterloo, 200 University Avenue West, Waterloo, Ontario N2L 3G1 Canada; Department of Pathology and Molecular Medicine, McMaster University, 1280 Main Street West, Hamilton, Ontario L8S 4K1 Canada; Neemo Inc, 1280 Main Street West, Hamilton, Ontario L8S 4K1 Canada

**Keywords:** Biofuel, *Clostridium*, Gene disruption, Intron, Metabolic engineering, Restriction-modification

## Abstract

**Background:**

*Clostridium pasteurianum* is one of the most promising biofuel producers within the genus *Clostridium* owing to its unique metabolic ability to ferment glycerol into butanol. Although an efficient means is available for introducing foreign DNA to *C. pasteurianum*, major genetic tools, such as gene knockout, knockdown, or genome editing, are lacking, preventing metabolic engineering of *C. pasteurianum*.

**Results:**

Here we present a methodology for performing chromosomal gene disruption in *C. pasteurianum* using the programmable lactococcus Ll.ltrB group II intron. Gene disruption was initially found to be impeded by inefficient electrotransformation of *Escherichia coli*-*C. pasteurianum* shuttle vectors, presumably due to host restriction. By assessing the ability of various vector deletion derivatives to electrotransform *C. pasteurianum* and probing the microorganism’s methylome using next-generation sequence data, we identified a new *C. pasteurianum* Type I restriction-methylation system, CpaAII, with a predicted recognition sequence of 5′-AAGNNNNNCTCC-3′ (N = A, C, G, or T). Following rescue of high-level electrotransformation via mutation of the sole CpaAII site within the shuttle vectors, we retargeted the intron to the *cpaAIR* gene encoding the CpaAI Type II restriction endonuclease (recognition site of 5′-CGCG-3′). Intron insertion was potentially hindered by low retrohoming efficiency, yet this limitation could be overcome by a procedure for enrichment of the intron insertion. The resulting Δ*cpaAIR* mutant strain was efficiently electrotransformed with M.FnuDII-unmethylated plasmid DNA.

**Conclusions:**

The markerless and plasmidless Δ*cpaAIR* mutant strain of *C. pasteurianum* developed in this study can serve as a general host strain for future genetic and metabolic manipulation. Further, the associated gene disruption protocol should not only serve as a guide for chromosomal gene inactivation studies involving mobile group II introns, but also prove invaluable for applying metabolic engineering strategies to *C. pasteurianum*.

## Background

Environmental and economic concerns surrounding the consumption of finite petroleum-based resources have led to the initiation of a rival biofuel industry, wherein cleaner and renewable sources of fuel are produced using biological catalysts [1]. Large-scale biofuel production is currently unfeasible, however, predominantly due to feedstock cost and availability [2]. Traditional feedstocks, such as corn and molasses, are readily fermentable, yet fluctuate drastically in price and compete with food supplies, rendering them unsustainable for biofuel production [3]. Cellulosic biomass, on the other hand, is abundant and economical, yet suffers from several bioprocessing drawbacks, including a requirement for costly enzymatic hydrolysis and tedious chemical pretreatment of raw substrates [4]. To be competitive with petrochemical processes, feedstocks for biofuel production must be cheap and abundant non-food resources that are readily fermented into value-added products. Crude glycerol, resulting from the production of biodiesel at a concentration of 1 kg of glycerol per 10 kg of biodiesel, is one of the few substrates satisfying these criteria [5–8]. Owing to the immense expansion of global biodiesel industries in recent years, crude glycerol has become a desirable and economical feedstock. However, microorganisms that can effectively dissimilate and ferment glycerol remain predominantly untapped.

*Clostridium pasteurianum* is a mesophilic, strictly anaerobic, Gram-positive bacterium that possesses the metabolic capacity to ferment glycerol as a sole source of carbon and energy, yielding a mixture of gases (hydrogen and carbon dioxide), acids (acetic and butyric), and alcohols (ethanol, butanol, and 1,3-propanediol) [9,10]. Of these products, butanol is a promising biofuel due to its resemblance to traditional gasoline with respect to physicochemical and fuel combustion properties. Although several microorganisms can metabolize glycerol, *C. pasteurianum* is the only species that converts glycerol to butanol, producing up to 17 g l^−1^ butanol [9], with a maximum yield of 0.36 g g^−1^ crude glycerol [11]. Biodiesel-derived glycerol requires only minor pretreatment to remove impurities [12] and allows fermentation performance comparable to that of refined glycerol [11,12]. The ability of *C. pasteurianum* to metabolize biodiesel-derived glycerol and its highly active butanol biosynthetic pathway make *C. pasteurianum* a bacterium of substantial biotechnological importance.

Several recent strategies have been employed in an attempt to alter the central metabolism of *C. pasteurianum* to enhance its productivity. Unfortunately, a lack of genetic tools has impeded metabolic engineering of *C. pasteurianum*, allowing only random chemical mutagenesis techniques [13,14]. It is clear that metabolic engineering will play a central role in the development of *C. pasteurianum* as an efficient industrial producer. To this end, an electroporation-mediated method of transformation was recently established [15], allowing gene transfer to *C. pasteurianum* with efficiencies of up to 10^4^ transformants μg^−1^ plasmid DNA. Such efficient plasmid transfer paves the way to rational metabolic engineering strategies, including gene disruption, knockdown, and overexpression techniques [16,17], none of which have been explored using *C. pasteurianum*. Gene disruption offers the most robust avenue for altering the expression of a native chromosomal gene or metabolic pathway. In *Clostridium*, the preferred tool for gene disruption is the ClosTron system, which has been adapted from TargeTron™ technology in *Escherichia coli* and exploits the retrohoming mechanism of bacterial group II introns [18–20]. Owing to the broad host range of group II introns, ClosTron-mediated gene disruptions have been performed in at least 11 species of *Clostridium* [18–25], leading to extensive metabolic engineering of solvent-producing clostridia [20,23,24].

Following our initial report of gene transfer to *C. pasteurianum*, we have observed that electrotransformation efficiency varies drastically between certain shuttle vectors [15]. Poor electrotransformation outcome was shown to be specific to vectors harboring lactococcal group II intron machinery. Restriction-modification (RM) systems are the most common cause of transformation recalcitrance in bacteria and potently inhibit plasmid transfer [17,26]. *C. pasteurianum* ATCC 6013 produces at least two active RM systems, CpaI (5′-GATC-3′) [27] and CpaAI (5′-CGCG-3′) [28], whereby restriction can be blocked using Dam (CpaI) and M.FnuDII (CpaAI) methylation, respectively [15]. Based on our preliminary observation, it is possible that *C. pasteurianum* expresses a third RM system that recognizes a specific nucleotide sequence within the clostridial gene disruption vectors. In this report, we show that group-II-intron-mediated chromosomal gene disruption in *C. pasteurianum* can be hindered by host restriction and low retrohoming efficiency. We demonstrate that overcoming these barriers leads to successful derivation of the first gene disruption mutant of *C. pasteurianum*. The developed system for chromosomal gene disruption will promote future metabolic engineering of *C. pasteurianum*.

## Results

### Shuttle vectors harboring Ll.ltrB intron machinery hinder electrotransformation of *Clostridium pasteurianum*

**Figure 1:**
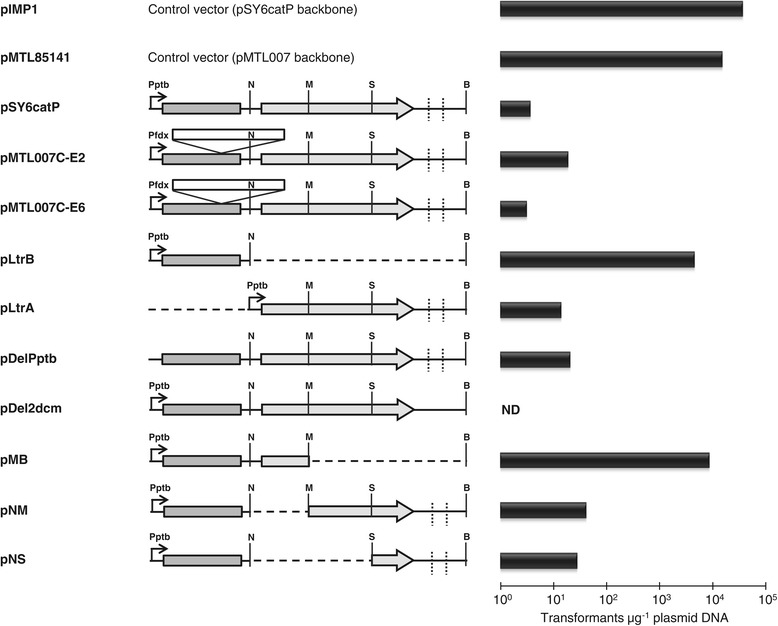
**Electrotransformation data demonstrating that** ***Clostridium pasteurianum*** **restricts a 932 bp SacII-BstAPI fragment within the** ***ltrA*** **gene of pSY6catP.** Only relevant vector regions corresponding to the intron components are shown and are depicted to scale. To allow alignment between constructs, deleted regions of vectors are represented as horizontal dashed lines and the *ermB* retrotransposition-activated marker (RAM; unshaded box) within plasmids pMTL007C-E2 and pMTL007C-E6 is shown above the Ll.ltrB intron. Shaded box: Ll.ltrB intron; unshaded box: *ermB* RAM; shaded arrow: *ltrA*; dashed line: Dcm recognition site; Pptb: *ptb* promoter (*Clostridium acetobutylicum*); Pfdx: *fdx* promoter (*Clostridium sporogenes*); ND: not detected. For vector pMB, the truncated *ltrA* gene is depicted as a box, rather than an arrow. Relevant restriction endonuclease recognition sites corresponding to BstAPI (B), MfeI (M), NheI (N), and SacII (S) are abbreviated using a single letter.

To attempt chromosomal gene disruption in *C. pasteurianum*, we first electrotransformed plasmid pSY6catP, which was developed for use in *Clostridium acetobutylicum* [15,20]. This vector harbors the Ll.ltrB intron and its cognate intron-encoded protein (IEP) gene, *ltrA*, both of which are transcribed from the same *C. acetobutylicum ptb* promoter within a pIMP1 vector backbone. Electrotransformation efficiencies of 3.7 × 10^4^ and 3.7 × 10^0^ transformants μg^−1^ plasmid DNA were obtained for pIMP1 and pSY6catP, respectively, indicating an inability of pSY6catP to transform *C. pasteurianum* (Figure 1). As the only difference between pIMP1 and pSY6catP is the presence of the intron machinery, we also attempted to transfer the ClosTron plasmid pMTL007C-E2, which expresses the same intron elements within a different, pMTL007-based vector backbone. Like pSY6catP, pMTL007C-E2 also yielded a poor electrotransformation efficiency of only 1.9 × 10^1^ transformants μg^−1^ plasmid DNA (Figure 1). Since pMTL007C-E2 possesses a different replication origin from pSY6catP (*repH* and *repL*, respectively), we constructed a *repL* derivative of pMTL007C-E2, named pMTL007C-E6, in order to allow direct comparison of the two *repL*-based intron-containing vectors, pMTL007C-E6 and pSY6catP. Like pMTL007C-E2 and pSY6catP, pMTL007C-E6 suffered from the same electrotransformation inhibition, generating only 3.2 × 10^0^ transformants μg^−1^ plasmid DNA, whereas the control vector, pMTL85141, gave 1.5 × 10^4^ transformants μg^−1^ plasmid DNA (Figure 1). Taken together, these outcomes demonstrate that shuttle vectors carrying the Ll.ltrB intron machinery are inhibitory to electrotransformation of *C. pasteurianum*, regardless of the vector backbone and replication origin employed.

### Inability to electrotransform Ll.ltrB-containing vectors is due to the presence of the *ltrA* gene

Since the Ll.ltrB intron and its cognate IEP gene, *ltrA*, are expressed from the same *ptb* promoter, we aimed to express the intron components independently in order to determine which element is responsible for electrotransformation inhibition. We constructed plasmids pLtrB and pLtrA, which individually express the intron and *ltrA* gene, respectively, with the use of the constitutive *C. acetobutylicum ptb* promoter. Upon transfer to *C. pasteurianum*, pLtrA hindered electrotransformation, giving an efficiency of 1.4 × 10^1^ transformants μg^−1^ plasmid DNA, whereas pLtrB generated 4.6 × 10^3^ transformants μg^−1^ plasmid DNA (Figure 1), indicating that electrotransformation inhibition is specific to the *ltrA* region of pSY6catP. Since plasmid pLtrA expresses a functional LtrA IEP product possessing maturase, endonuclease, and reverse transcriptase activities [29], low electrotransformation efficiency could be the result of toxicity [30]. To test this hypothesis, we constructed pDelPptb, in which the −35 and −10 signals of the *ptb* promoter within pSY6catP were deleted. The resulting plasmid should not produce intron RNA or a protein product corresponding to the IEP. Despite deletion of the promoter, pDelPptb failed to improve electrotransformation efficiency and generated only 2.1 × 10^1^ transformants μg^−1^ plasmid DNA (Figure 1), implying that low electrotransformation efficiency was not associated with toxicity of the intron and IEP.

In light of these results, we speculated that a specific nucleotide sequence within the *ltrA* gene could be targeted by an uncharacterized RM system in *C. pasteurianum*. Plasmid pLtrA contains two *E. coli* Dcm (5′-CCWGG-3′; W = A or T) restriction recognition sites immediately downstream of the *ltrA* coding sequence, which are not found in the control vector, pIMP1. Since *E. coli*-*C. pasteurianum* shuttle vector preparations destined for *C. pasteurianum* electrotransformation are first methylated in a Dcm^+^*E. coli* host strain (ER1821), all such plasmids would be methylated at both Dcm recognition sites (5′-CmCWGG-3′). Certain methylated Dcm sites have been shown to potently inhibit electrotransformation of *Clostridium thermocellum* [31] and *Clostridium ljungdahlii* [32]. Therefore, we examined if Dcm methylation contained within the *ltrA* gene region was responsible for the decline in electrotransformation efficiency by constructing plasmid pDel2dcm, in which both Dcm recognition sites were mutated. Plasmid pDel2dcm failed to generate any detectable transformants (Figure 1), indicating that Dcm methylation is not responsible for the reduced electrotransformation efficiency of pSY6catP.

### *Clostridium pasteurianum* restricts a 334 bp region of the *ltrA gene* region and restriction can be overcome by extensive codon modification

In a first attempt to identify any unknown restriction recognition sequences within plasmid pLtrA, three constructs were prepared by replacing various-sized restriction fragments in the *ltrA* gene and its downstream region with a 48 bp stuffer fragment. The sizes of these restriction fragments were 1,661 bp (pMB), 1,434 bp (pNS), and 688 bp (pNM) (Figure 1). Upon electrotransformation of the three resulting vectors, only pMB gave an improved electrotransformation efficiency (8.8 × 10^3^ transformants μg^−1^ plasmid DNA), whereas pNS and pNM yielded efficiencies of 2.8 × 10^1^ and 4.2 × 10^1^ transformants μg^−1^ plasmid DNA, respectively (Figure 1). This result indicates that the putative restriction endonuclease recognition sequence is contained within a 932 bp SacII-BstAPI restriction fragment of pSY6catP (corresponding to 493 bp of the *ltrA* coding region and 439 bp downstream of the *ltrA* gene).

**Figure 2:**
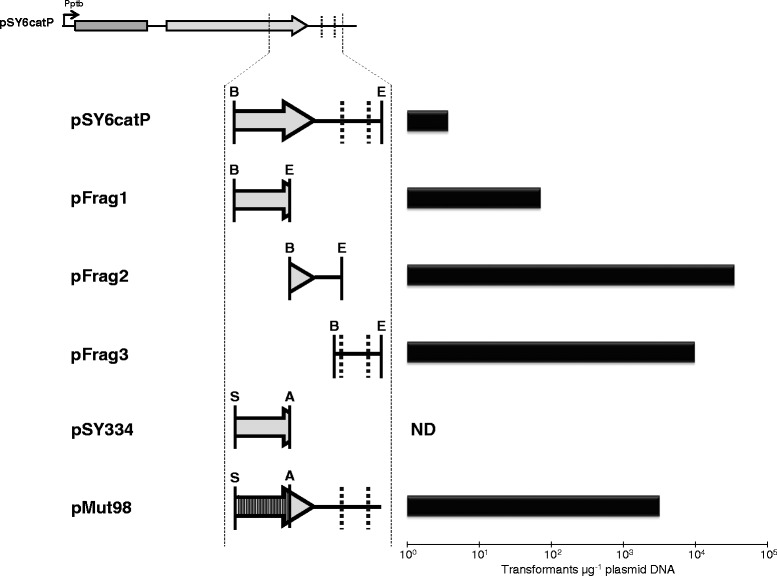
**Electrotransformation data demonstrating that** ***Clostridium pasteurianum*** **restricts a 334 bp region within the** ***ltrA*** **gene sequence of pSY6catP.** The 932 bp SacII-BstAPI region of pSY6catP (see Figure 1) is enlarged to better show relevant vector components. Point mutations within the *ltrA* coding sequence are depicted as vertical bands. Enlarged vector components are depicted to scale. Shaded box: Ll.ltrB intron; shaded arrow: *ltrA*; dashed line: Dcm recognition site; Pptb: *ptb* promoter (*C. acetobutylicum*); ND: not detected. Relevant restriction endonuclease recognition sites corresponding to AatII (A), BglII (B), EcoO109I (E), and SacII (S) are abbreviated using a single letter.

To reduce the size of the putative vector region responsible for electrotransformation inhibition, we constructed another three vectors in which a 1,332 bp BglII-EcoO109I restriction fragment was replaced with one of the three different regions in the 932 bp SacII-BstAPI restriction fragment. Of the initial 932 bp region, plasmids pFrag1, pFrag2, and pFrag3 possess fragments of sizes 339 bp, 338 bp, and 305 bp, respectively (Figure 2). Approximately 20 to 30 bp overlap was contained between PCR fragments to ensure the unknown restriction recognition site would be represented in its entirety. Of these three constructs, only pFrag1 showed a significant reduction in electotransformation efficiency (7.1 × 10^1^ transformants μg^−1^ plasmid DNA) compared to the control vector (pIMP1; 3.7 × 10^4^ transformants μg^−1^ plasmid DNA), whereas pFrag2 and pFrag3 yielded efficiencies of 3.5 × 10^4^ and 9.7 × 10^3^ transformants μg^−1^ plasmid DNA, respectively (Figure 2). This result reduced the inhibitory region of pSY6catP from 932 bp to 339 bp. To further verify that the 339 bp region of the *ltrA* coding sequence is responsible for inhibition of electrotransformation of *C. pasteurianum*, we aimed to insert the detrimental sequence into a control vector that is able to electrotransform *C. pasteurianum* at a high efficiency. Thus, we constructed plasmid pSY334 by subcloning 334 bp of the 339 bp inhibitory fragment from pSY6catP into pMTL85141 using SacII and AatII restriction sites. Plasmid pMTL85141 consistently electrotransforms *C. pasteurianum* with efficiencies on the order of 10^3^to 10^4^ transformants μg^−1^ plasmid DNA. As expected, pSY334 failed to generate any transformants upon several electrotransformation attempts (Figure 2), suggesting potent activity of the uncharacterized RM system in *C. pasteurianum*.

To overcome the uncharacterized restriction barrier, we attempted to mutate the unknown restriction recognition sequence in the 334 bp inhibitory region of pSY6catP. Since this region is contained within the *ltrA* gene sequence, our efforts were limited to silent mutations, which would conserve the amino acid sequence and yield a functional *ltrA* gene product. Consequently, we silently mutated 98 bp spanning 83 codons within the 334 bp *ltrA* coding sequence, generating plasmid pMut98, with the greatest number of consecutive nucleotides left unaltered following mutagenesis being 5 bp. Since most RM recognition sequences are greater than or equal to 6 bp, we envisioned that the unknown restriction recognition site would be mutated within pMut98. As expected, pMut98 electrotransformed *C. pasteurianum* with an efficiency of 3.2 × 10^3^ transformants μg^−1^ plasmid DNA, an increase of approximately three orders of magnitude compared to unmodified pSY6catP (Figure 2). We then subcloned the mutated 334 bp *ltrA* coding region into plasmids pMTL007C-E2 and pMTL007C-E6, yielding pMTLCP-E2 and pMTLCP-E6, respectively. Similar to pMut98, codon modification led to significantly increased electrotransformation efficiencies for both pMTLCP-E2 (1.9 × 10^3^ transformants μg^−1^ plasmid DNA) and pMTLCP-E6 (2.7 × 10^3^ transformants μg^−1^ plasmid DNA), compared to 1.9 × 10^1^ transformants μg^−1^ plasmid DNA for pMTL007C-E2 and 3.2 × 10^0^ transformants μg^−1^ plasmid DNA for pMTL007C-E6 (Figure 1).

### Methylome analysis unveils a unique restriction-modification system in *Clostridium pasteurianum* (CpaAII) that restricts a single 5′-AAGNNNNNCTCC-3′ site within pSY6catP

Using single molecule real-time (SMRT) sequencing data, it is possible to probe an organism’s unique DNA methylation profile (methylome). To determine a putative recognition sequence of the uncharacterized RM system, we utilized existing *C. pasteurianum* SMRT genome sequencing data generated using the RS II analyzer [33]. Due to current technical restraints in SMRT methylome analysis, only m6A residues could be detected, as identification of m5C residues requires more extensive sequence coverage. Surprisingly, methylome analysis revealed a previously unidentified methylation motif possessing a putative recognition sequence of 5′-AAGNNNNNCTCC-3′, which we have designated CpaAII. A single CpaAII recognition site exists within the 334 bp of the *ltrA* gene shown to inhibit electrotransformation of *C. pasteurianum*. To further verify the CpaAII recognition sequence, we constructed plasmid pDelCpaAII, in which the unique CpaAII recognition site was mutated by introducing only three silent point mutations within the coding sequence of *ltrA*. Similar to pMut98, with 98 silent mutations and a high electrotransformation efficiency of 3.2 × 10^3^ transformants μg^−1^ plasmid DNA, pDelCpaAII generated a 432-fold increase in electrotransformation efficiency (1.6 × 10^3^ transformants μg^−1^ plasmid DNA) compared to unmodified pSY6catP (3.7 × 10^0^ transformants μg^−1^ plasmid DNA). Conversely, we aimed to create a unique CpaAII recognition site by introducing only two point mutations within pMTL85141, which does not contain any CpaAII recognition sites and electrotransforms *C. pasteurianum* efficiently. The resulting plasmid, pCpaAII, failed to electrotransform *C. pasteurianum*, further verifying the existence of the new RM system.

We analyzed all other *E. coli*-*C. pasteurianum* shuttle vectors utilized in this work and only pMTL007C-E2 and pMTL007C-E6, both harboring the lactococcal group II intron machinery, were found to possess a CpaAII site. The CpaAII site within these vectors is identical to that in pSY6catP, as both are located within the *ltrA* gene. Unexpectedly, both pMTL007C-E2 and pMTL007C-E6 were found to possess an additional CpaAII site, which was reconfirmed by Sanger sequencing, within the erythromycin (*ermB*) retrotransposition-activated-marker (RAM) region. To assess the effect of the additional CpaAII site on electrotransformation, we used plasmids pMTLCP-E2 and pMTLCP-E6, in which the detrimental CpaAII site within the *ltrA* coding sequence is mutated yet the additional site within the *ermB* RAM is left unaltered, to electrotransform *C. pasteurianum*. As reported above, pMTLCP-E2 and pMTLCP-E6 generated relatively high electrotransformation efficiencies of 1.9 × 10^3^ transformants μg^−1^ plasmid DNA and 2.7 × 10^3^ transformants μg^−1^ plasmid DNA, respectively, suggesting that the additional CpaAII site is not subject to restriction by *C. pasteurianum*.

### Generation of an intron-mediated gene disruption mutant of *Clostridium pasteurianum*

**Figure 3:**
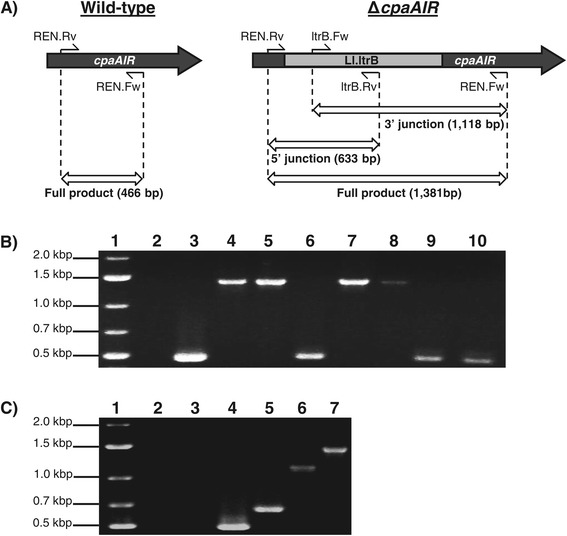
**Identification and verification of Δ** ***cpaAIR*** **mutant colonies of** ***Clostridium pasteurianum*** **. A)** Schematic diagram depicting primer annealing sites and expected PCR products of wild-type cells (left) and Δ*cpaAIR* mutant cells (right). Insertion of the Ll.ltrB intron into the *cpaAIR* gene leads to a 915 bp increase in size of the full-length PCR product generated using primers flanking the 176a intron insertion site (REN.Rv + REN.Fw). Both 5′ and 3′ gene-intron junction PCR products can be detected in Δ*cpaAIR* mutant cells using primer sets REN.Rv + ltrB.Rv and ltrB.Fw + REN.Fw, respectively. **B)** Colony PCR screening of gene disruption enrichment colonies for presence of intron insertion by amplification of the full-length product. Lane 1: marker; lane 2: no template control; lane 3: wild-type, non-recombinant *C. pasteurianum* colony; lanes 4 to 10: gene disruption enrichment colonies; lanes 4, 5, 7, and 8: positive colonies; lanes 6, 9, and 10: negative colonies. **C)** Further genomic verification of a single positive Δ*cpaAIR* mutant colony by amplification of all three PCR products depicted in Figure 3A (5′ junction, 3′ junction, and full product). A wild-type *C. pasteurianum* colony was included as a control for all three PCR primer sets. Lane 1: marker; lanes 2 to 4: wild-type colony; lanes 5 to 7: Δ*cpaAIR* mutant colony; lanes 2 and 5: 5′ junction; lanes 3 and 6: 3′ junction; lanes 4 and 7: full product.

Since plasmid pMut98 afforded a substantial improvement in electrotransformation efficiency compared to pSY6catP, we used it as a *C. pasteurianum* gene disruption vector. In addition, we replaced the *C. acetobutylicum ptb* promoter controlling intron transcription with the promoter from the *C. pasteurianum* thiolase gene. The resulting vector, pSYCP, was then used to target nucleotide position 176 within the antisense strand (176a) of the putative *cpaAIR* gene (corresponding to locus tag CP6013_2592), which was identified within the draft genome sequence of *C. pasteurianum* ATCC 6013 [GenBank accession number JPGY01000000] [33] and encodes the CpaAI Type II restriction endonuclease. Consistent with restriction analyses [15,28], The Restriction Enzyme Database (REBASE) [34] predicts a recognition sequence of 5′-CGCG-3′ for the putative *cpaAIR* gene product. We selected the *cpaAIR* gene for gene disruption since the predicted 176a insertion site generated a high predicted insertional score (7.3), gene disruption is unlikely lethal, and the resulting mutant should prove useful for future genetic and metabolic engineering applications by abolishing the requirement for methylation of plasmid DNA prior to electrotransformation [22,35]. The retargeted pSYCP-cpaAIR plasmid was electroporated to *C. pasteurianum* and transformants were selected using thiamphenicol. Transformant colonies were first screened for insertion of the intron within the *cpaAIR* coding sequence, resulting in an insertion of 915 bp, using two gene-specific primers flanking the predicted 176a intron insertion site (Figure 3A). Of 28 screened colonies, all possessed the wild-type PCR product without the intron insertion. However, both gene-intron junctions could be detected in several pSYCP-cpaAIR transformant colonies using one gene-specific and one intron-specific primer (data not shown), signifying successful intron insertion had occurred. The resulting mosaic colonies were presumed to be comprised of a mixture of wild-type and intron-disrupted mutant cells, potentially due to low retrohoming efficiency (J. Perutka, personal communication). To enrich and isolate the mutant cells, mosaic colonies were subcultured in liquid 2 × YTG medium containing thiamphenicol every 12 hours for a total of 5 days, or 10 transfers, followed by rescreening of the resulting colonies for the intron insertion using two gene-specific primers. Of seven colonies screened, four possessed the desired intron insertion (Figure 3B). Finally, one such positive colony was selected and used for additional PCR verification by amplifying both gene-intron junctions to ensure proper intron insertion and orientation (Figure 3C).

In another attempt for gene disruption, we constructed plasmids pMTLCP-E2-cpaAIR and pMTLCP-E6-cpaAIR, which possess an erythromycin RAM allowing direct selection of cells containing an intron insertion. Thiamphenicol-resistant transformants harboring pMTLCP-E2-cpaAIR or pMTLCP-E6-cpaAIR were restreaked onto erythromycin-containing 2 × YTG agar plates to select for intron disruption mutants. Upon restreaking 24 transformant colonies, no erythromycin-resistant colonies were able to grow, signifying unsuccessful insertion of the RAM-containing intron into the *cpaAIR* gene of *C. pasteurianum*. Therefore, we recommend plasmid pSYCP, containing a markerless Ll.ltrB intron, with our developed enrichment method for performing chromosomal gene disruptions in *C. pasteurianum*.

### Curing of intron donor plasmid pSYCP-cpaAIR

Prior to phenotypic characterization of the resulting Δ*cpaAIR* mutant, we attempted to cure the pSYCP-cpaAIR intron donor plasmid. Based on previous reports involving plasmid curing in *Clostridium*, we employed a method based on repeated subculturing in non-selective growth medium [22,23]. A single Δ*cpaAIR* mutant colony was grown in liquid 2 × YTG medium and the seed culture was used to inoculate fresh growth medium every 12 hours for a total of three or seven successive transfers. The resulting cells were serially diluted and plated onto non-selective 2 × YTG agar. A total of 36 colonies from the three-transfer and seven-transfer methods were picked and restreaked onto both non-selective and thiamphenicol-containing 2 × YTG agar plates. A total of 18 and 33 thiamphenicol-sensitive colonies, corresponding to curing efficiencies of 50% and 92%, were identified in the three- and seven-transfer approaches, respectively. A total of four thiamphenicol-sensitive colonies were subjected to further confirmation of plasmid curing based on their sensitivity to thiamphenicol and their inability to generate a plasmid-borne PCR product upon conducting colony PCR using primers frag3.BglII.S + frag3.EcoO109I.AS (data not shown).

### Characterization of the *Clostridium pasteurianum* Δ*cpaAIR* gene disruption mutant

**Figure 4:**
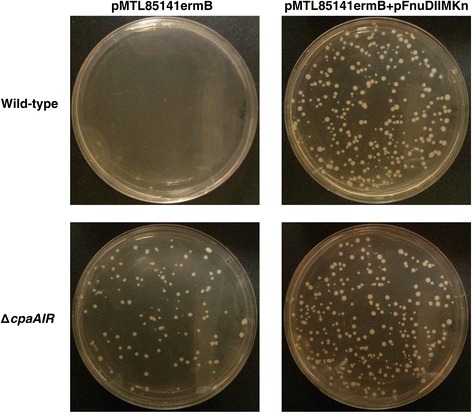
**Electrotransformation results demonstrating successful electrotransformation of Δ** ***cpaAIR*** **gene disruption cells with M.FnuDII-unmethylated plasmid pMTL85141ermB.** Wild-type cells (top row) and Δ*cpaAIR* gene disruption cells (bottom row) of *Clostridium pasteurianum* were electroporated separately with M.FnuDII-unmethylated (left column) and M.FnuDII-methylated (right column) plasmid pMTL85141ermB. M.FnuDII methylation was achieved *in vivo* using an *Escherichia coli* strain harboring pMTL85141ermB and pFnuDIIMKn. Varying volumes of electrotransformation outgrowth cell suspensions were plated to give approximately equal numbers of transformants between electrotransformations. Hence, the number of transformant colonies shown does not allow for a direct comparison of electrotransformation efficiency.

The resulting Δ*cpaAIR* gene disruption mutant should not produce a functional CpaAI Type II restriction endonuclease and, therefore, should be efficiently transformed with plasmid DNA lacking methylation by the FnuDIIM methyltransferase (5′-m5CGCG-3′) [15]. To test this phenotype, we used the plasmid-cured Δ*cpaAIR* mutant strain to assess its capacity to be electrotransformed with both M.FnuDII-unmethylated and M.FnuDII-methylated plasmid substrates (Figure 4). We first verified that M.FnuDII unmethylated plasmid pMTL85141ermB, a dual erythromycin- and thiamphenicol-selectable derivative of pMTL85141 [15], fails to electrotransform wild-type *C. pasteurianum* (Figure 4, top left panel) due to CpaAI restriction, whereas M.FnuDII-methylated pMTL85141ermB electrotransforms efficiently (Figure 4, top right panel; electrotransformation efficiency of 1.1 × 10^4^ transformants μg^−1^ plasmid DNA). We next attempted to electrotransform M.FnuDII-unmethylated plasmid pMTL85141ermB to Δ*cpaAIR* mutant cells and, as expected, pMTL85141ermB (Figure 4, bottom left panel; electrotransformation efficiency of 9.6 × 10^2^ transformants μg^−1^ plasmid DNA) electrotransformed at a level comparable to M.FnuDII-methylated pMTL85141ermB (Figure 4, bottom right panel; electrotransformation efficiency of 2.3 × 10^3^ transformants μg^−1^ plasmid DNA). Successful electrotransformation of methylation-deficient plasmid DNA indicates inactivation of the *cpaAIR* Type II endonuclease in the Δ*cpaAIR* mutant strain. Note that electrotransformation efficiency of Δ*cpaAIR* cells was more than an order of magnitude lower than wild-type *C. pasteurianum*, which we have observed in two independent electrotransformation experiments. We are uncertain of the reason for the reduced electrotransformation efficiency of the Δ*cpaAIR* mutant. However, electrotransformation efficiency of the Δ*cpaAIR* mutant strain is still comparable to many other clostridial procedures [36–39], generating 10^2^ to 10^3^ transformants μg^−1^ plasmid DNA.

## Discussion

Genetic manipulation techniques, particularly chromosomal gene disruption, have found widespread use in the solventogenic clostridia, permitting rational metabolic engineering approaches and the generation of mutant strains with enhanced metabolic capabilities [17]. While *C. pasteurianum* has recently been identified as a promising industrial producer due to its unique metabolic capabilities, it has suffered from a lack of available genetic tools for extensive strain improvement. Here, we further apply our recent DNA electrotransformation protocol to conduct chromosomal gene disruption in *C. pasteurianum* with concurrent identification and characterization of a new *C. pasteurianum* RM system. This biotechnological development is expected to pave the way towards genetic and metabolic engineering of this microorganism for a wider range of industrial applications. Technically, we show that the ability to perform group-II-intron-mediated chromosomal gene disruptions in *C. pasteurianum* is limited by host restriction and low-level intron retrohoming efficiency, and overcoming these obstacles leads to successful derivation of *C. pasteurianum* mutant strains.

By assessing transformability of several plasmid deletion derivatives of pSY6catP (Figures 1 and 2) and probing the methylome of *C. pasteurianum* using SMRT genome sequencing data, we have concluded that the inability of intron-containing vectors for chromosomal gene disruption to efficiently electrotransform *C. pasteurianum* is due to restriction by a new RM system, designated as CpaAII. Based on methylation motifs predicted from methylome analysis, we propose a recognition sequence of 5′-AAGNNNNNCTCC-3′ for the CpaAII RM system. The proposed CpaAII recognition sequence is indicative of Type I RM systems, which are widespread in bacteria and recognize nucleotide sequences comprised of 4 to 8 degenerate (N) residues flanked by short (2 to 5 bp) defined sequences [34]. Further, all Type I RM systems characterized to date encode m6A-specific methyltransferases [40], as we have proposed for CpaAII. Finally, a single Type I RM system is annotated in the draft genome sequence of *C. pasteurianum* ATCC 6013 [33] and encodes the three host specificity domains, corresponding to restriction (*hsdR*; locus tag CP6013_1662), modification (*hsdM*; locus tag CP6013_1663), and specificity (*hsdS*; locus tag CP6013_1664), that are typical characteristics of Type I RM systems [40].

All vector derivatives with a significantly reduced ability to electrotransform *C. pasteurianum* in this study were found to possess at least one CpaAII recognition sequence. In the case of pSY6catP, mutation of the single CpaAII recognition site within the *ltrA* gene sequence restored electrotransformation to a level comparable to control vectors lacking lactococcal group II intron elements. Conversely, only one of the two CpaAII restriction recognition sequences within ClosTron plasmids pMTL007C-E2 and pMTL007C-E6 was found to negatively affect electrotransformation efficiency of *C. pasteurianum*. A possible explanation for this outcome is that the actual CpaAII recognition sequence may differ slightly from the proposed motif predicted by methylome analysis in this study. It is likely that the actual CpaAII recognition sequence is more stringent than the predicted 5′-AAGNNNNNCTCC-3′ motif, as only one of the two CpaAII recognition sequences within pMTL007C-E2 and pMTL007C-E6 was subject to restriction. For example, the recognition sequence of the Type I RM system from *C. perfringens* ATCC 13124, 5′-CACNNNNNRTAAA-3′ (R = A or G), is similar in structure to that of the predicted CpaAII enzyme (5′-AAGNNNNNCTCC-3′), yet contains a partially-degenerate 5 bp element at the 3′ end, rather than a defined 4 bp element in the case of CpaAII. Increasing the coverage of SMRT sequencing data (our ongoing approach) could potentially resolve this apparent discrepancy.

Having overcome host restriction of shuttle vectors harboring the lactococcal *ltrA* gene, we used pMut98, which contains no CpaAII recognition sites, as the basis for deriving our chromosomal gene disruption vector. However, retrohoming efficiency of the intron proved to be too low to isolate a homogeneous gene disruption mutant directly from pSYCP-cpaAIR transformant colonies, as most colonies were found to represent a heterogeneous mixture of wild-type and mutant cells. Mixed-genotype transformant colonies have been described in other intron-based gene disruption studies [24]. This outcome is not specific to the *cpaAIR*(176a) insertion site employed in this study, as we have observed mixed-genotype colonies using predicted target sites of three other chromosomal genes in *C. pasteurianum* (data not shown). As such, retrohoming efficiency of the Ll.ltrB intron appears to be lower in *C. pasteurianum* compared to other solventogenic clostridia [18–20], as gene disruption mutants are typically identified and isolated following transfer of the intron donor plasmid. The use of a selectable RAM inserted into domain IV of the intron [18,19] within vectors pMTL007C-E2/pMTLCP-E2 and pMTL007C-E6/pMTLCP-E6 was not able to enhance selection and isolation of true intron disruption colonies, presumably due to a further decrease in retrohoming efficiency resulting from the presence of cargo DNA within the intron sequence [18]. Thus, to isolate a homogeneous gene disruption colony, we employed an enrichment procedure by subculturing a heterogeneous transformant colony in selective liquid medium to promote intron insertion. Following our enrichment protocol, approximately half of the screened colonies possessed the desired intron insertion (Figure 3), suggesting its effectiveness in increasing retrohoming efficiency of the Ll.ltrB intron in *C. pasteurianum*.

Plasmid curing has proven to be an essential and often laborious aspect of clostridial strain engineering efforts. Several clostridial host-vector systems have been shown to produce plasmids that exhibit strong segregational stability in the absence of selection. This has led to the development of strategies involving induction of plasmid instability through the use of negative-selectable markers (such as *pyrF* [41]) and antisense RNA targeted to the plasmid replication protein [42]. We have shown that efficient plasmid curing in *C. pasteurianum* does not require artificial induction methods, as plasmids based on the common *repL* replication origin can be efficiently cured by subculturing cells in non-selective growth medium for a total of only three successive transfers (approximately two days). This outcome contrasts other reported clostridial plasmid curing procedures, which often require up to seven successive transfers to cure replicative intron donor plasmids [22,25].

The Δ*cpaAIR* mutant strain developed in this study does not harbor plasmids nor antibiotic resistance markers, as it was constructed without the use of a RAM-containing intron, and should prove advantageous for future genetic and metabolic engineering efforts by abolishing the requirement for M. FnuDII methylation of shuttle vectors prior to electrotransformation. Note that shuttle vectors transforming Δ*cpaAIR* mutant cells still require CpaI methylation, which can be readily performed using Dam^+^*E. coli* strains for plasmid propagation. Analogous restriction-negative mutants have also been produced in *C. acetobutylicum* [35] and *Clostridium cellulolyticum* [22] through disruption of the genes encoding the Cac824I and CceI Type II RM systems, respectively. Our gene disruption system should prove to be broadly applicable to any non-essential gene within the genome of *C. pasteurianum*, permitting that a viable intron insertion site can be identified. Since metabolic engineering approaches often involve disruption of multiple genes and metabolic pathways, strains with multiple markerless intron insertions can be envisioned using our strategy by employing iterative rounds of intron retargeting, electrotransformation, enrichment, and plasmid curing. In fact, a recent report has detailed intron-mediated disruption of up to five genes in *C. acetobutylicum* [43]. Since the Δ*cpaAIR* mutant constructed in this report is the first *C. pasteurianum* mutant strain obtained using group II intron technology, we recommend using the *cpaAIR*(176a) target site and plasmid pSYCP-cpaAIR as a control for future intron-mediated gene disruption studies. The *cpaAIR*(176a) insertion site generated a predicted insertional score of 7.3 using a Ll.ltrB insertion site prediction algorithm (TargeTronics, LLC, Austin, Texas, United States) and, therefore, we recommend selecting sites of equal or greater score for disruption of genes in *C. pasteurianum* using the Ll.ltrB group II intron. It is expected that the intron donor plasmid and associated gene disruption methodologies detailed herein will add to the expanding genetic toolkit available for *C. pasteurianum* and lead to rewarding metabolic engineering efforts involving this important biotechnological bacterium.

## Conclusions

In this work, we have developed and utilized a gene disruption plasmid (pSYCP) and intron enrichment strategy to generate a mutant strain of *C. pasteurianum* deficient in CpaAI restriction. The resulting Δ*cpaAIR* strain is devoid of plasmids and antibiotic resistance markers, and does not require M.FnuDII methylation of plasmid DNA substrates to achieve efficient electrotransformation, making it an ideal host background for metabolic engineering applications. This is the first report of gene disruption and targeted genetic manipulation of *C. pasteurianum* and represents an important divergence from previously employed random chemical mutagenesis methods.

## Methods

### Bacterial strains, plasmids, and oligonucleotides

Bacterial strains and plasmids employed in this work are listed in Table 1 and oligonucleotide sequences are given in Table 2. *E. coli* DH5α was utilized for vector construction and cloning purposes and ER1821[pFnuDIIMKn] for methylation of *E. coli*-*C. pasteurianum* shuttle vectors destined for *C. pasteurianum* [15]. Vectors pIMP1 [44] and pSY6 [20] were kindly provided by Professor Terry Papoutsakis (University of Delaware, Newark, Delaware, United States) and Professor Sheng Yang (Chinese Academy of Sciences, Shanghai, China). Plasmid pMTL85141 [45] and the ClosTron vector, pMTL007C-E2 [18], were kindly shared by Professor Nigel Minton (University of Nottingham, Nottingham, United Kingdom). Oligonucleotides and gBlocks were synthesized by Integrated DNA Technologies (IDT, Coralville, Iowa, United States). Oligonucleotides were prepared at the 25 nm scale using standard desalting. Custom gene synthesis was performed by Bio Basic Inc. (Markham, Ontario, Canada).

**Table 1:** **Strains and plasmids employed in this study**

**Strains or plasmids**	**Relevant characteristics**	**Source or reference**
Strains		
*Escherichia coli* DH5α	F^−^ *endA1 glnV44 thi-1 recA1 relA1 gyrA96 deoR nupG φ80dlacZΔM15 Δ(lacZYA-argF)U169, hsdR17(r*_*K*_^*−*^ *m*_*K*_^*+*^ *), λ* ^*−*^	Lab stock
*Escherichia coli* ER1821	F^−^ *endA1 glnV44 thi-1 relA1? e14* ^*−*^ *(mcrA* ^*−*^ *) rfbD1? spoT1? Δ(mcrC-mrr)114::IS10*	Lab stock; New England Biolabs
*Clostridium pasteurianum* ATCC 6013	Wild-type	American Type Culture Collection
*Clostridium pasteurianum* Δ*cpaAIR*	Disruption mutant generated by inserting the Ll.ltrB intron into position 176a of the *cpaAIR* gene encoding the CpaAI RENase	This study
Plasmids		
pFnuDIIMKn	M.FnuDII methyltransferase plasmid for methylation of *E. coli*-*C. pasteurianum* shuttle vector s (Km^R^ ; p15A ori)	[15]
pIMP1	*E. coli-Clostridium* shuttle vector (Ap^R^; ColE1 ori; Em^R^; *repL* ori)	[44]
pMTL85141	*E. coli-Clostridium* shuttle vector (Cm^R^/Tm^R^; ColE1 ori; *repL* ori)	[45]
pMTL85141ermB	*E. coli-Clostridium* shuttle vector (Cm^R^/Tm^R^; Em^R^; ColE1 ori; *repL* ori)	[15]
pSY6catP	*E. coli-Clostridium* shuttle vector expressing the Ll.ltrB intron and *ltrA* IEP from the *Clostridium acetobutylicum ptb* promoter (Ap^R^ ; Cm^R^/Tm^R^; ColE1 ori; *repL* ori)	[15]
pMTL007C-E2	ClosTron vector expressing the Ll.ltrB intron with RAM and *ltrA* IEP from the *Clostridium sporogenes fdx* promoter (Cm^R^/Tm^R^; ColE1 ori; *repH* ori; Em^R^ RAM)	[18]
pMTL007C-E6	*repL* derivative of pMTL007C-E2	This study
pltrB	*ltrA*-deletion derivative of pSY6catP	This study
pltrA	Ll.ltrB-deletion derivative of pSY6catP	This study
pDelPptb	Derived by deleting the −35 and −10 signals of the *C. acetobutylicum ptb* promoter from plasmid pSY6catP	This study
pDel2dcm	Derived by mutating the two *E. coli* Dcm restriction recognition sites downstream of the *ltrA* coding sequence within plasmid pSY6catP	This study
pMB	Derived by replacing a 1,661 bp MfeI + BstAPI restriction fragment of pSY6catP with a 48 bp stuffer fragment	This study
pNM	Derived by replacing a 688 bp NheI + MfeI restriction fragment of pSY6catP with a 48 bp stuffer fragment	This study
pNS	Derived by replacing a 1,434 bp NheI + SacII restriction fragment of pSY6catP with a 48 bp stuffer fragment	This study
pFrag1	Derived by replacing a 1,332 bp BglII + EcoO109I restriction fragment of pSY6catP with a 589 bp *ltrA* region	This study
pFrag2	Derived by replacing a 1,332 bp BglII + EcoO109I restriction fragment of pSY6catP with a 363 bp *ltrA* region	This study
pFrag3	Derived by replacing a 1,332 bp BglII + EcoO109I restriction fragment of pSY6catP with a 574 bp *ltrA* region	This study
pSY334	Derived by subcloning a 334 bp SacII + AatII fragment of the *ltrA* coding sequence into plasmid pMTL85141	This study
pMut98	pSY6catP derivative possessing 98 silent mutations in the *ltrA* coding sequence	This study
pMTLCP-E2	Derived by subcloning a 1,427 bp MscI + AclI restriction fragment of pMut98 into plasmid pMTL007C-E2	This study
pMTLCP-E6	Derived by subcloning a 1,427 bp MscI + AclI restriction fragment of pMut98 into plasmid pMTL007C-E6	This study
pDelCpaAII	Deletion of the unique CpaAII recognition site within pSY6catP by introducing three silent point mutations	This study
pCpaAII	Introduction of a unique CpaAII recognition site within pMTL85141 by introducing two point mutations	This study
pSYCP-cpaAIR	Derived by replacing the *ptb* promoter of pMut98 with a *thl* promoter and targeting the Ll.ltrB intron to position 176a of the *cpaAIR* gene	This study
pMTLCP-E2-cpaAIR	Targeting construct of plasmid pMTLCP-E2 for disruption of the *cpaAIR* gene at position 176a	This study
pMTLCP-E6-cpaAIR	Targeting construct of plasmid pMTLCP-E6 for disruption of the *cpaAIR* gene at position 176a	This study

Ap^R^: ampicillin resistant; Cm^R^: chloramphenicol resistant; Em^R^: erythromycin resistant; Km^R^: kanamycin resistant; Tm^R^: thiamphenicol resistant.

**Table 2:** **Oligonucleotides employed in this study**

**Oligonucleotide**	**Sequence (5′-3′)**
ltrB.NheI.S	*CTAGC*GCTATATGCGTTGATGCAATTTCTATGCACTCGTAGTAGTCTGAGAAG*GCATATG*
ltrB.BstAPI.AS	*ATGC*CTTCTCAGACTACTACGAGTGCATAGAAATTGCATCAACGCATATAGC*G*
ltrA.XhoI.S	TTTCTA*CTCGAG*GCGTTGATGCAATTTCTATGCACTC
ltrA.BstAPI.AS	GGCATCAGAGCAGATTGTACTGAG
del-Pptb.S	*GGG*GTTAATCATTTAACATAGATAATTAAATAGTAAAAGGGAGTGTCGAGATATC*C*
del-Pptb.AS	*TCGAG*GATATCTCGACACTCCCTTTTACTATTTAATTATCTATGTTAAATGATTAAC*CCC*
MfeI/BstAPI.S	*AATTG*ATTTAGTAATTTCTATAAGCAGGTTAGCTGTAAAACTAGCAGTAGCAC*GCATATG*
MfeI/BstAPI.AS	*ATGC*GTGCTACTGCTAGTTTTACAGCTAACCTGCTTATAGAAATTACTAAAT*C*
NheI/MfeI.S	*CTAGC*ATTTAGTAATTTCTATAAGCAGGTTAGCTGTAAAACTAGCAGTAGCAC*C*
NheI/MfeI.AS	*AATTG*GTGCTACTGCTAGTTTTACAGCTAACCTGCTTATAGAAATTACTAAAT*G*
NheI/SacII.S	*CTAGC*ATTTAGTAATTTCTATAAGCAGGTTAGCTGTAAAACTAGCAGTAGCAC*CCGC*
NheI/SacII.AS	*GG*GTGCTACTGCTAGTTTTACAGCTAACCTGCTTATAGAAATTACTAAAT*G*
frag1.BglII.S	GGGATATGATATACGAGTAAGG*AGATCT*GG
frag1.EcoO109I.AS	AGTATT*AGGCCCT*GACGTCCCACATAATTCACAACATTTAGC
frag2.BglII.S	AACAGG*AGATCT*GCTAAATGTTGTGAATTATGTGGGACGTC
frag2.EcoO109I.AS	TACTCT*AGGCCCT*GGAGACCCCACACTACCATCG
frag3.BglII.S	TCGCCA*AGATCT*CGATGGTAGTGTGGGGTCTCC
frag3.EcoO109I.AS	GTGCCACCTGACGTCTAAGAAACC
3′SOE.S	TGGGAAATGGCAATGATAGCGAAAC
SOE.EcoO109I.AS	ATAGGCGTATCACG*AGGCCCT*TTC
gBlock.BglII.S	CGAGTAAGG*AGATCT*GGAACGATAAAACG
CpaAII-anneal.S	*CTAGA*GTCGACGTCACGCGTCCAAGGAGATCTCCAGGCCTGCAGACATGC*A*
CpaAII-anneal.AS	*AGCTT*GCATGTCTGCAGGCCTGGAGATCTCCTTGGACGCGTGACGTCGAC*T*
SYCP.gBlock.S	GGAGGTCAATCTATGAAAATGCGATTAAGC
SYCP.gBlock.AS	CTTTCGTTTCGTTCCCATAGGTTCTCC
MTLCP.REN-HindIII.S	GTATTTA*AAGCTT*ATAATTATCCTTAAATTTCTTAAAAGTGCGCC
REN.Fw	CTACTTGAGGTCTAGGACTTCTATCT
REN.Rv	ACAGATAGGCCATTAAAGGTATTCA
ltrB.Fw	CCAACGCGTCGCCACGTAATAAAT
ltrB.Rv	ATGGGAACGAAACGAAAGCGATGC

Italics: relevant restriction endonuclease recognition sequences.

### Growth and maintenance conditions

*E. coli* strains were cultivated aerobically at 37°C in lysogeny broth (LB) and recombinant derivatives were selected, when necessary, with ampicillin (100 μg ml^−1^), chloramphenicol (30 μg ml^−1^), or kanamycin (30 μg ml^−1^) (Sigma-Aldrich; St. Louis, Missouri, United States). Antibiotic levels were reduced by half for selection of *E. coli* strains harboring two vectors. *C. pasteurianum* strains were grown anaerobically at 37°C in 2 × YTG medium (16 g l^−1^ tryptone, 10 g l^−1^ yeast extract, 5 g l^−1^ glucose, and 4 g l^−1^ sodium chloride, pH 6.3) (Sigma-Aldrich; St. Louis, Missouri, United States) within an anaerobic containment chamber (Plas-Labs; Lansing, Michigan) containing an atmosphere of 5% CO_2_, 10% H_2_, and 85% N_2_. Strict anaerobic conditions were maintained and monitored through the use of a palladium catalyst fixed to the heater of the chamber, removal of oxygen from growth medium via autoclaving, and addition of resazurin (1 mg l^−1^) to all solid and liquid media preparations. Recombinant *C. pasteurianum* strains were selected, when necessary, with 10 μg ml^−1^ thiamphenicol or 25 μg ml^−1^ erythromycin. Recombinant *E. coli* and *C. pasteurianum* were stored frozen in 15% glycerol at −80°C (both species) or as sporulated colonies on solidified 2 × YTG agar plates (*C. pasteurianum*).

### DNA isolation, manipulation, and electrotransformation

Plasmid DNA was extracted from *E. coli* and purified using an EZ-10 Spin Column Plasmid DNA Miniprep Kit (Bio Basic, Inc.; Markham, Ontario, Canada). Intact, high molecular weight *C. pasteurianum* genomic DNA was extracted from a 60 ml culture (OD_600_ 0.5-0.7) by first washing cells in 40 ml of a buffer containing 25 mM potassium phosphate, pH 7.0, and 6 mM MgSO4, followed by resuspension in 15 ml of the same buffer supplemented with 50% sucrose and 200 μg/ml lysozyme (Sigma-Aldrich; St. Louis, Missouri, United States) [46,47]. After anaerobic incubation at 37°C for 45 minutes, genomic DNA was extracted from 1.0 to 5.0 ml samples of protoplast suspension using a DNeasy Blood and Tissue Kit from Qiagen (Valencia, California, United States). Due to the high nuclease content of clostridia, hypertonic sucrose (50% w/v) was added to buffer ATL during cell lysis [47]. Eluted genomic DNA was treated with 100 μg/ml RNase A prior to additional purification using a Genomic DNA Clean & Concentrator kit from Zymo Research (Irvine, California, United States).

DNA restriction fragments and PCR products were purified directly or from agarose gels using an EZ-10 Spin Column DNA Gel Kit (Bio Basic, Inc.; Markham, Ontario, Canada). Vector construction was carried out according to standard procedures [48]. Restriction enzymes, Standard *Taq* DNA Polymerase, Phusion High-Fidelity DNA Polymerase, and Quick Ligation Kit were purchased from New England Biolabs (Whitby, Ontario, United States). All commercial enzymes and kits were used according to the manufacturer’s instructions. Electrotransformation of *C. pasteurianum* was performed as previously described [15].

### Vector construction

Plasmid pMTL007C-E6 was constructed from pMTL007C-E2 by ligation of a 878 bp AscI + FseI digestion fragment of pMTL85141 containing the *repL* replication module with a 7,300 bp product of pMTL007C-E2 resulting from digestion with the same restriction enzymes.

pLtrB was constructed by digesting pSY6catP with NheI + BstAPI, extracting the resulting 6,131 bp fragment, and ligating it with complementary oligos ltrB.NheI.S + ltrB.BstAPI.AS that had been annealed to generate compatible NheI and BstAPI restriction ends. Complementary oligonucleotides were mixed in equimolar amounts, heated to 95°C in a water bath, and allowed to anneal by disconnecting the power source from the water bath. For construction of pLtrA, the entire 3,426 bp Ll.ltrB-*ltrA* intron region was removed from pSY6catP using XhoI + BstAPI digestion and replaced with a 2,398 bp PCR product containing only the *ltrA* coding sequence, generated using primers ltrA.XhoI.S + ltrA.BstAPI.AS. The resulting product was digested with XhoI + BstAPI and ligated with the 5,072 bp pSY6catP vector backbone to place *ltrA* under transcriptional control of the *ptb* promoter. Plasmid pDelPptb was derived from pSY6catP by replacement of the 131 bp SmaI + XhoI digestion product containing the *C. acetobutylicum ptb* promoter with a 56 bp stuffer fragment lacking −35 and −10 promoter signals derived by annealing oligonucleotides del-Pptb.S + del-Pptb.AS. Ligation-proficient SmaI and XhoI restriction ends were generated upon successful annealing of complementary oligonucleotides. pDel2dcm was derived from pSY6catP by replacing a 924 bp SacII + BstAPI restriction fragment with the same 924 bp sequence in which the two Dcm sites were mutated by two single-base-pair mutations. The Dcm deletion fragment was synthesized by Bio Basic, Inc. (Markham, Ontario, Canada), digested with SacII + BstAPI, and ligated into the corresponding sites of pSY6catP. The three restriction fragment deletion constructs, pMB, pNM, and pNS, were prepared by digesting pSY6catP with MfeI + BstAPI, NheI + MfeI, and NheI + SacII, respectively, and annealing the resulting vector backbones with the respective annealed oligonucleotide pairs, MfeI/BstAPI.S + MfeI/BstAPI.AS (pMB), NheI/MfeI.S + NheI/MfeI.AS (pNM), and NheI/SacII.S + NheI/SacII.AS (pNS).

To construct pFrag1, pFrag2, and pFrag3, a 1,332 bp BglII + EcoO109I restriction fragment was removed from pSY6catP and replaced with a 589 bp (primers frag1.BglII.S + frag1.EcoO109I.AS), 363 bp (primers frag2.BglII.S + frag2.EcoO109I.AS), or 574 bp (primers frag3.BglII.S + frag3.EcoO109I.AS) PCR product, respectively, corresponding to various products of the *ltrA* coding region of pSY6catP. To construct pSY334, a 334 bp SacII + AatII fragment of the *ltrA* coding sequence was subcloned into the corresponding sites of pMTL85141. To mutate the unknown restriction recognition sequence within the inhibitory 334 bp region of the *ltrA* coding sequence, a 655 bp gBlock was synthesized possessing 98 silent mutations in which 83 codons were altered. A 731 bp PCR product containing the 3′ *ltrA* coding sequence and downstream region, and possessing 25 bp overlap with the mutated gBlock, was amplified using primers 3′SOE.S + SOE.EcoO109I.AS. The PCR product was loaded on a 1.0% agarose gel (Bio Basic, Inc.; Markham, Ontario, Canada), stabbed with a micropipette tip, and used as template along with 5 ng of the purified gBlock in a splicing by overlap extension (SOE) PCR by cycling for 10 cycles prior to adding primers gBlock.BglII.S + SOE.EcoO109I.AS and cycling for 25 additional cycles. The resulting product was digested with BglII + EcoO109I and ligated with pSY6catP that had been digested with the same restriction endonucleases to generate pMut98. Plasmids pMTLCP-E2 and pMTLCP-E6 were constructed by subcloning a 1,427 bp MscI + AclI restriction fragment of pMut98 into the corresponding sites of pMTL007C-E2 and pMTL007C-E6, respectively.

To mutate the CpaAII recognition site of pSY6catP using three silent point mutations, a gBlock was synthesized in which codons 521 (AGU → UCU; Ser) and 523 (GCU → GCC; Ala) were mutated within the *ltrA* coding sequence. In a manner similar to pMut98, the mutated gBlock was fused with the same 731 bp 3′ *ltrA* product using SOE PCR. The resulting product was digested with BglII + EcoO109I and ligated with BglII- and EcoO109I-digested pSY6catP to yield plasmid pDelCpaAII. Conversely, a unique CpaAII restriction recognition sequence was generated within pMTL85141 by first annealing complementary oligonucleotides CpaAII-anneal.S + CpaAII-anneal.AS. The resulting annealed product, possessing cohesive XbaI + HindIII ends, was ligated into the corresponding sites of pMTL85141 to give plasmid pCpaAII.

Ll.ltrB intron design was performed using the computer algorithm developed by TargeTronics, LLC (Austin, Texas, United States). The insertion site with the highest predicted insertion score splicing into the antisense strand was selected corresponding to nucleotide position 176 (score of 7.3) of the CpaAI restriction endonuclease gene, *cpaAIR*. Plasmid pMut98 was used as the basis for a *C. pasteurianum* TargeTron gene disruption vector. For retargeting pMut98, a 572 bp gBlock fragment was synthesized possessing mutations in the IBS, EBS2, and EBS1d intron regions corresponding to position 176a of the *cpaAIR* gene. The retargeted gBlock was designed with a constitutive *C. pasteurianum* thiolase promoter controlling transcription of the intron and *ltrA* gene. The gBlock fragment was PCR-amplified using primers SYCP.gBlock.S + SYCP.gBlock.AS, digested with BamHI + BsrGI, and ligated into the corresponding sites of pMut98 to generate pSYCP-cpaAIR. To retarget the ClosTron vectors pMTLCP-E2 and pMTLCP-E6 to the *cpaAIR* gene of *C. pasteurianum*, primers MTLCP.REN-HindIII.S and SYCP.gBlock.AS were used to amplify the gBlock targeted to the CpaAI endonuclease. The resulting 384 bp PCR product was digested with HindIII + BsrGI and ligated into the corresponding sites of pMTLCP-E2 and pMTLCP-E6 to give pMTLCP-E2-cpaAIR and pMTLCP-E6-cpaAIR, respectively.

### Single molecule real-time genome sequencing and methylome analysis

SMRT sequencing was performed on intact, purified genomic DNA from *C. pasteurianum* according to a previous report [33]. SMRT reads were assembled by the Biosciences Division at Oak Ridge National Laboratory (Oak Ridge, Tennessee, United States) and the resulting assembly was used as a reference genome for methylome analysis, which was carried out by Pacific Biosciences (Menlo Park, California, United States).

### Group-II-intron-mediated gene disruption, enrichment, and screening

To isolate a chromosomal gene disruption mutant, plasmid pSYCP-cpaAIR was first electrotransformed into *C. pasteurianum* and transformants were selected using 10 μg ml^−1^ thiamphenicol. Mosaic colonies containing both wild-type and intron insertion cells were identified with two separate PCRs using primers ltrB.Fw + REN.Fw for one chromosome-intron junction and primers ltrB.Rv + REN.Rv for the adjacent junction. One positive colony was selected for enrichment of the intron disruption by repeated subculturing in selective growth medium. Briefly, a sporulated colony was heat-shocked in 10 ml of 2 × YTG medium, cooled on ice, and supplemented with 10 μg ml^−1^ thiamphenicol (Sigma-Aldrich; St. Louis, Missouri, United States). Following approximately 24 hours of growth, 0.5 ml was used to inoculate a tube of 10 ml 2 × YTG containing 10 μg ml^−1^ thiamphenicol. This process was repeated every 12 hours for a total of 10 transfers, at which time serial dilutions were plated onto nonselective 2 × YTG agar. To identify a homogeneous gene disruption colony, colony PCR was performed on enrichment colonies using two gene-specific primers flanking the intron insertion site (REN.Fw + REN.Rv).

### Curing Δ*cpaAIR* cells of plasmid pSYCP-cpaAIR

To cure Δ*cpaAIR* disruption cells of the pSYCP-cpaAIR intron donor plasmid, a single Δ*cpaAIR* disruption colony was heat-shocked in 10 ml 2 × YTG medium without selection. Once exponential-phase growth was observed, 0.5 ml was used to inoculate a new tube of 10 ml 2 × YTG. This process was repeated every 12 hours for a total of three transfers, at which time serial dilutions were plated onto nonselective 2 × YTG agar. Colonies were screened for the absence of plasmid pSYCP-cpaAIR by restreaking onto both non-selective and selective (10 μg ml^−1^ thiamphenicol) 2 × YTG agar plates. For thiamphenicol-sensitive colonies, plasmid loss was further confirmed by lack of colony PCR amplification using primers Frag3.BglII.S + Frag3.EcoO109I.AS and absence of growth in liquid 2 × YTG medium containing 10 μg ml^−1^ thiamphenicol.

## Abbreviations

bp: base pair
IEP: Intron-encoded protein
PCR: polymerase chain reaction
RAM: Retrotransposition-activated marker
RM: Restriction-modification
SMRT sequencing: Single molecule real time sequencing
SOE PCR: Splicing by overlap extension PCR
